# Chinese Medicine Bu Xu Hua Yu Recipe for the Regulation of Treg/Th17 Ratio Imbalance in Autoimmune Hepatitis

**DOI:** 10.1155/2015/461294

**Published:** 2015-04-21

**Authors:** Lei Wang, Huihui Du, Yibo Liu, Lingtai Wang, Xiong Ma, Wei Zhang

**Affiliations:** ^1^Department of Hepatology, Shanghai University of Traditional Chinese Medicine, Longhua Hospital, No. 725 South Wan Ping Road, Shanghai, China; ^2^Shanghai University of Traditional Chinese Medicine, Shuguang Hospital, Shanghai, China; ^3^Department of Gastroenterology, Renji Hospital, Shanghai Institute of Digestive Disease, Shanghai Jiao Tong University School of Medicine, China

## Abstract

*Objectives*. The aim of this study is researching the role of the Regulatory T cell (Treg)/T helper cell-17 (Th17) cell ratio imbalance in the pathogenesis of autoimmune hepatitis (AIH) and the use of the “Bu Xu Hua Yu” recipe in the treatment of AIH.* Materials and Methods*. Sixty adult male C57/BL6 mice were divided into six different groups. *α*-Galcer was injected abdominally for production of the animal models. Liver function tests, histological examinations, liver tissue Regulatory T cell, and T helper cell-17 levels tests were carried out. TGF-*β*1, IL-10, IL-17, and expression of mRNA and protein levels of Foxp3 and ROR-*γ*t were also assessed.* Results*. Bu Xu Hua Yu method increased the levels of Regulatory T cell, IL-10, and the expression of Foxp3 (*P* < 0.05) in mice liver tissues. Furthermore, there were decreases in the levels of T helper cell-17, IL-17, and expression of ROR*γ*t mRNA and protein (*P* < 0.05). The ratio of Treg/Th17 was increased (*P* < 0.05).* Conclusion*. Mice with AIH have a Treg/Th17 ratio imbalance. Bu Xu Hua Yu method was able to restore the cellular balance of Treg/Th17 through the regulation of the expression of ROR*γ*t and Foxp3 and can play an important role in the treatment of AIH.

## 1. Introduction

Autoimmune hepatitis (AIH) is a type of inflammatory liver disease whose etiology is not fully understood and with no standard treatment protocol. As of now, single dose prednisone or low dose prednisone plus azathioprine is the main treatment protocol for AIH, with alleviation of symptoms achieved in 60% to 80% of cases [1]. However, it must be noted that 50% to 86% of patients witness a recurrence of the disease after stopping medications, while 13% of patients stop the treatment too early due to the severity of the side effects and 9% of patients' situation will deteriorate, even though they are administered medications according to guidelines [2]. The pathophysiology of AIH is relatively complicated and the incidence of the disease is closely related to genetics, autoimmune antibodies, environmental factors, and imbalance of the immune system. In these last years, studies have shown that the activation of autoimmune responsive T cells and B cells is an important step in the pathophysiology of the disease and the loss of balance in the levels of these two cells is essential to the pathogenesis of AIH. However, these assumptions have, as of now, not been proven.

The use of Chinese medicine for the treatment of autoimmune hepatitis has proven its merits in mainland China and is able to make up for the problems encountered with western medicine. The need for better understanding the mechanisms of these treatment protocols has put a spotlight onto those types of studies and is bound to become a major converging point. We made use of the Bu Xu Hua Yu principle of immunological regulation, which can stand for restoring deficiency and removing blood clots (exact content of this treatment is given in Table 2), combined with Ursodeoxycholic acid capsules (Losan pharma GmbH, Germany, specification: 250 mg × 25 pills) to prevent AIH and this treatment protocol showed results which were similar to those obtained with prednisone combined with azathioprine. Furthermore, the Chinese medicine protocol was better apt at controlling the symptoms of the disease. Following the new developments in the study of AIH animal model, this study made use of *α*-Galcer to induce C57BL/6 mice AIH animal model. *α*-Galcer is able to cause NKT induced liver damage and infiltration of CD4+ lymphocytes in liver tissues as seen in classic presentations of AIH as has been previously reported in other studies by Biburger and Tiegs and by Matsumoto et al. [3, 4]. At the same time, modeling is rendered simple and time for modeling is relatively short. *α*-Galcer hepatitis model has differences in individual susceptibility gene; C57BL/6 mice showed higher *α*-Galcer hepatitis susceptibility. We, therefore, combined what we already know about the mechanism of AIH and early stages of clinical studies to investigate the function of T cells, especially the role of regulatory T cells (Treg)/T helper cells-17 (Th17) abnormalities in the etiology of AIH, with especial emphasis on the mechanism of the Bu Xu Hua Yu treatment method.

## 2. Materials and Methods

This study was approved by the Institutional Animal Care and Use Committee of Shanghai University of Traditional Chinese Medicine. The registration number for our approved experiment was 11007 and the following measures were taken to ameliorate animal suffering.

1. The person taking care of the experimental animals during the study is very experienced in this type of work, has very good animal handling skills, and took very good care of all animals involved in this study on a daily basis.
2. All experiments of the animals were done after inducing anesthesia so as to decrease any pain that the animals might have felt.
3. All animals were euthanized after the experiment was carried out.
4. After they were introduced in our laboratory settings, all animals were given individual cages and the cages were cleaned on a very regular basis.

### 2.1. Choice of Experimental Animal and Grouping

Sixty male C57/BL6 mice of SPF grade and aged between 4 and 6 weeks were chosen. The mice were obtained from Shanghai Si Lai Ke Experimental Animal Limited Liability Company (license number: SCXK 2007-0005, Shanghai, China). The animals were bred at Shanghai University of Traditional Chinese Medicine Affiliated Long Hua Hospital's SPF grade laboratory animal room. We made use of randomized block design to randomly separate the 60 mice into control group (K), model group (M), steroids group (X), Chinese medicine lose dose group (D), Chinese medicine moderate dose group (Z), and Chinese medicine high dose group (G) with each group having 10 mice. A preexperiment was used, by the same group of researchers, to study the viability of this study before engaging in a larger scale study with more mice.

### 2.2. Main Materials and Reagents

FACS flow cytometry was products of BD company (Becton, Dickinson and Company, East Rutherford, New Jersey, United States); CD4+CD25+ regulatory T cell mice staining kit mainly contains 3 types of antibody, namely, FTTC-anti-mouse-CD4+, APC-anti-mouse-CD25, and PE-Cy5-anti-mouse-Foxp3. The Th17 cell staining kit (PE Rat Anti-Mouse IL-17A, APC IFN-*γ* Anti-Mouse, FTTC-anti-mouse-CD3e, and PerCP Rat Anti-Mouse CD4) was obtained from BD Company. The TGF-*β*1, IL-10, and IL-17 Elisa reagent kit was obtained from Shanghai Ao Biological Company (Shanghai, China); the antibodies were obtained from R&D systems (Minneapolis, USA). The Trizol reagent was bought from Invitrogen Company (California, USA). Foxp3, ROR*γ*t, and the reference GAPDH primers were synthesized by Shanghai ShineGene Biotechnology Companies (Shanghai, China); cDNA synthesis kit and PCR amplification kit were provided by TaKaRa Co. Biological Engineering (Dalian, China); protein extraction kit was provided by the Bestbio Biotechnology Companies (Shanghai, China); Mouse Anti-Human Foxp3 (2A11GG) polyclonal antibody and Rabbit Anti-Human ROR*γ*t (H-190) polyclonal antibody were purchased from Santa-Cruz Company (Santa Cruz Biotechnology, Inc.). Reference antibody Histone H3.1 (Ab-10) was obtained from SignalWay Antibody (Maryland, USA).

### 2.3. Experiment Method

#### 2.3.1. Drug Intervention and Preparation of AIH Mice Model

*Bu Xu Hua Yu Drug Preparation*. A mouse (20 g) is more or less equivalent to a human adult (approximately 60 Kg). For the normal daily dosage, the following are required for the Bu Xu Hua Yu recipe: dried* Rehmannia* roots 0.05 g,* Angelica* 0.04 g,* Astragalus* 0.05 g, red peony root 0.05 g, Chuanxiong 0.03 g, and* Sedum sarmentosum* 0.10 g.

*Dosage*. Dosage was calculated according to the “Estimating the Safe Starting Dose in Clinical Trials for Therapeutics in Adult Healthy Volunteers” guidelines. Low dose group received a dose equivalent to 10 times the normal clinical dose of the normal adult and one time the dose for mice; medium dose group received a dose equivalent to 20 times the normal clinical dose of a normal adult and 2 times the dose for mice; high dose group received a dose equivalent to 40 times the normal clinical dose of a normal adult and 4 times the dose for mice; steroid control group received prednisone 0.1 mg/(mice·day). Mice in the model control group and in the blank control group were given isovolumetric normal saline solution 0.2 mL/mice.

Each group was first given the respective medications for 7 days and on the 7th day, except for the control group; the five other groups all received an intra-abdominal injection of 2 *μ*g/10 g *α*-Galcer (Enzo Life Sciences).

*Histological Analysis of the Liver of Mice with AIH*. The liver is fixed with 10% neutral formalin and embedded in paraffin wax and sliced. The slides were stained with hematoxylin and eosin. The degree of inflammation of the liver was graded as follows: grade 0 = normal liver tissue; grade 1 = mild infiltration of inflammatory cells with rare hepatic cells necrosis; grade 2 = medium damage of hepatic cells accompanied by infiltration of inflammatory cells and regional hepatic cell necrosis; grade 3 = wide range inflammatory cells infiltration in portal area and hepatic lobules accompanied with wide range hepatic cells necrosis.

*Biochemical Indexes*. Ten to twelve hours after injection of the modeling agent, blood was drawn from the mice's eyeball, was kept at 4°C for 2 hours, and was centrifuged at 3000 rpm for 15 mins. After centrifugation, the serum sample was collected and was analyzed for alanine aminotransferase (ALT), aspartate aminotransferase (AST), and alkaline phosphatase (AKP). All experiments were done strictly following the instructions of Nanjing Jian Cheng Ke Ji (Nanjing, China) who provided us with the liver function test kit.

### 2.4. Flow Cytometry Was Used to Detect the Number of Treg Cells and Th17 Cells in the Hepatic Tissue

#### 2.4.1. Treg Cell

We used monocytes extracted from lymphocytes of the liver; the cell concentration was set at 1 × 10^7^/mL. A 100 *μ*L hepatic monocytes suspension was added to 0.25 *μ*L FITC anti-mice CD4 monoclonal antibody, 0.3 *μ*L APC-anti-mice CD25 + monoclonal antibody, and 2.5 *μ*L PE-anti mice Foxp3 monoclonal antibody incubation. FACS flow cytometry was used to detect the number of Treg cells and the content of Foxp3 in liver.

#### 2.4.2. Th17 Cell

We used monocytes extracted from lymphocytes of the liver; the cell concentration was set at 1 × 10^7^/mL. A 100 *μ*L hepatic monocytes suspension was added to PerCP labeled anti-CD4, CD3e, after incubation with broken membrane agent, intracellular cytokine staining, and FITC labeled IL-17A 0.5 *μ*g incubation. FACS flow cytometry was used to detect Th17 cytokines, IL-17, and IFN-*γ* which are secreted by CD4+ cells.

### 2.5. ELISA Method for the Detection of Liver Homogenate IL-10, IL-17, and TGF-*β*1 Levels

The experiment was carried out according to the strict instructions of the ELISA kit.

### 2.6. RT-PCR Was Used to Analyze the Expression of Foxp3 mRNA and ROR*γ*t mRNA

(1) Primer synthesis: the specific sequence is as follows:

 Foxp3: Forward Prime 5′-ACCCAGGAAAGACAGCAACC-3′, Reverse Primer 5′-CTCGAAGACCTTCTCACAACCA-3′, length of products: 107 bp
 ROR*γ*t: Forward Primer 5′-CCATTGACCGAACCAGCC-3′, Reverse Primer 5′-TCTGCTTCTTGGACATTCGG-3′, length of products: 111 bp
 Gapdh: Forward Primer 5′-TGTGTCCGTCGTGGATCTGA-3′, Reverse Primer 5′-TCTGCTTCTTGGACATTCGG-3′, length of products: 77 bp.

(2) Extraction of total RNA and RT-PCR procedure: 50–100 mg tissue was collected from each group; Trizol kit was used to extract total RNA, after using the spectrophotometer at 260/280 nm in the examination of the quality and purity of total RNA. Using the cDNA synthesis kit we performed transcription of mRNA to cDNA. We then used cDNA corresponding specific primers for PCR amplification with the real-time PCR. 20 *μ*L reaction volume includes Premix Ex TaqTM (2x) 10 *μ*L, upstream and downstream primers 0.4 *μ*L, Taq Probe 0.8 *μ*L, Rox Reference Dye (50x) 0.4 *μ*L, cDNA 2 *μ*L, and DEPC water 6 *μ*L. PCR procedure was done as follows: 95°C for 30 s after the initial hot-start, followed by 45 PCR cycles at 95°C for 5 s, annealing temperature of 60°C for 30 s.

(3) Extraction of 10 *μ*L PCR reaction product: 10 *μ*L PCR reaction product was extracted and placed on a 2% agarose gel electrophoresis for 50 min and the image acquisition is done by image analyzer. Using glyceraldehyde-3-phosphate dehydrogenase (Gapdh) reference as the benchmark, we performed semiquantitative analysis. Real-time PCR results are shown as follows: 2-ΔΔCt (2-ΔΔCt = RQ) represents the target genes relative to GAPDH. Higher values for 2-ΔΔCt correspond to higher levels of gene expression and smaller values correspond to lesser levels of gene expression.

### 2.7. Western Blot Technique Used to Measure the Expression of Foxp3 and ROR*γ*t Protein in Hepatic Tissue

After 50–100 mg of hepatic tissue was homogenized, tissue proteins were extracted; BCA method was used for measuring the concentration of protein levels and for dilution. Sodium dodecyl sulfate polyacrylamide gel electrophoresis was carried out and the protein was transferred to the PVDF film; Ponceau S staining was used for the examination of the strip film; 1 × TBST buffer skim milk closed 1 h, 1 : 100 Foxp3, ROR*γ*t, and 1 : 200 reference antibody were separately added, respectively, and were incubated overnight at 4°C; phosphate buffer was added after washing the membrane with horseradish peroxidase-labeled IgG; electrochemiluminescence color was obtained through X-ray exposure, developing and fixing was done and Gel-Pro analysis software was used to locate and analyze the strip.

### 2.8. Statistical Analysis

All data obtained in this study was analyzed using the SPSS16.0 software. Data for each group is represented in the form ($\overset{-}{X}\pm S$); multisamples groups were compared with single factor analysis of variance (one-way ANOVA). Skewness distribution was analyzed using nonparametric test. *P* < 0.05 was considered to be statistically significant; *P* < 0.01 was considered to be statistically highly significant.

## 3. Results

### 3.1. Tissue Histology Examination of AIH Mouse Model and of Different Groups after Drug Usage

**Figure 1:**
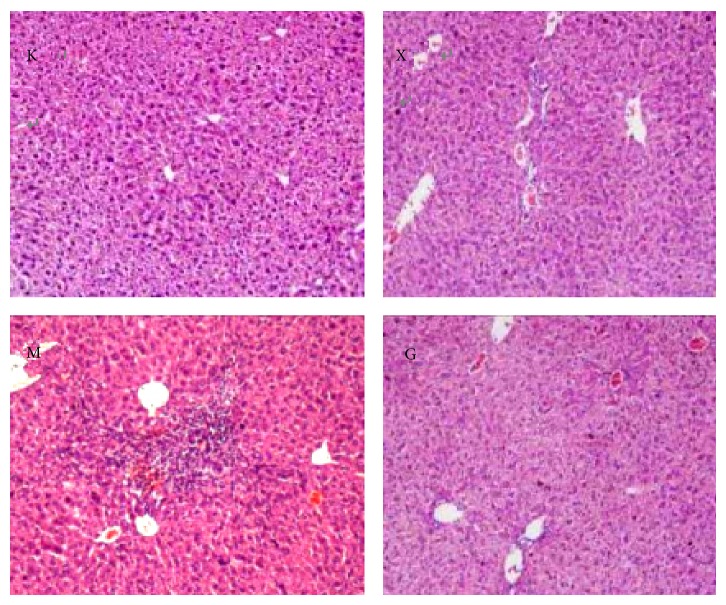
The figure shows the histological analysis for control group (K), model group (M), steroids group (X), and Chinese medicine high dose group (G). Histological analysis for each group.

Hepatic tissue of mouse after hematoxylin-eosin staining shows that after induction with *α*-Galcer, the C57BL/6 mice showed varying degrees of pathological changes of grade 1 to grade 2 inflammation; the portal area and its surroundings showed obvious infiltration of inflammatory cells; when compared to the model group, steroids group and the Chinese medicine moderate dose group showed signs of mild inflammation, with remission of the infiltration of inflammatory cells; blank control group showed almost normal hepatic tissue, as shown in Figure 1.

### 3.2. Result for Liver Function Indexes

**Figure 2:**
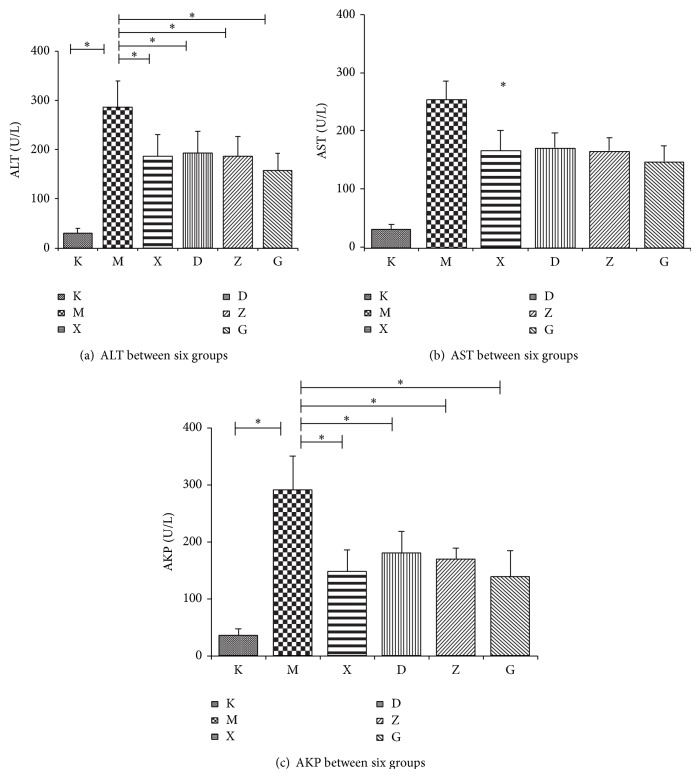
The figure shows the level of ALT, AST, and AKP for each of the six groups.

Except for the blank control group, the liver function indexes were increased by different amounts in all the other groups. After treatment of steroids group and the different Chinese medicine groups, the liver function indexes all witnessed decreases which were statistically significant when compared with the control group (*P* > 0.05). However, the difference between the steroids and Chinese medicine groups and the difference between the different Chinese medicine groups were not statistically significant (*P* > 0.05). This shows that the Chinese medicine was able to protect the liver against AIH and the results were on par with steroids. The results are shown in Table 1 and Figure 2 (Figure 2(a) for ALT levels, Figure 2(b) for AST levels, and Figure 2(c) for AKP levels).

### 3.3. Analysis of Results for Hepatic Lymph Cell Treg (CD4+CD25+Foxp3+) Cell and Th17 (CD3+CD4+IL-17+IFN-*γ*−) Cell

**Figure 3:**
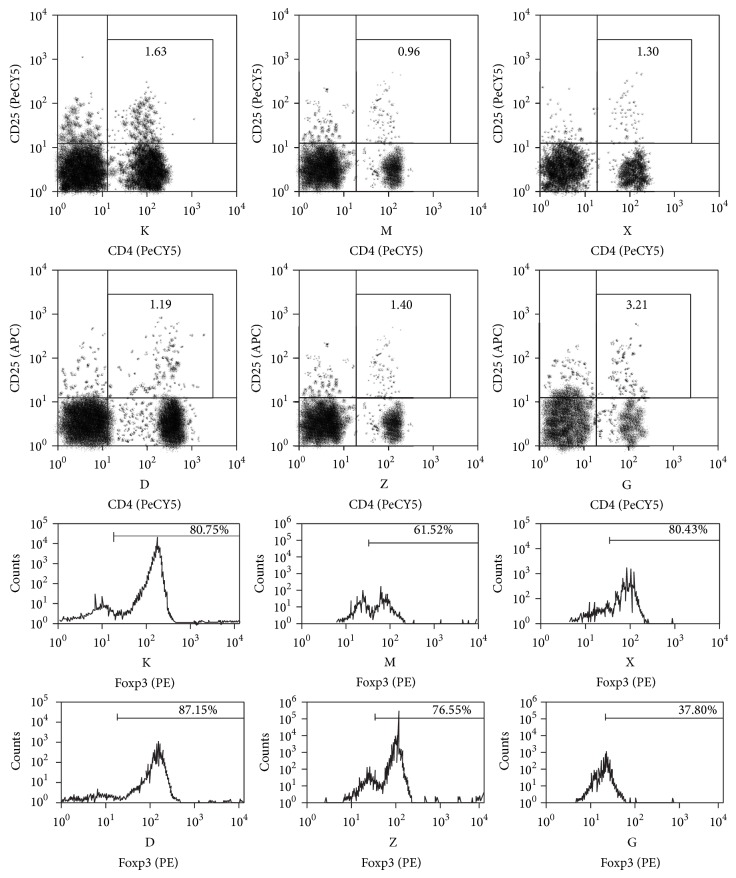
Treg (CD4+CD25+Foxp3+) FACS hepatic tissue for each group.

**Figure 4:**
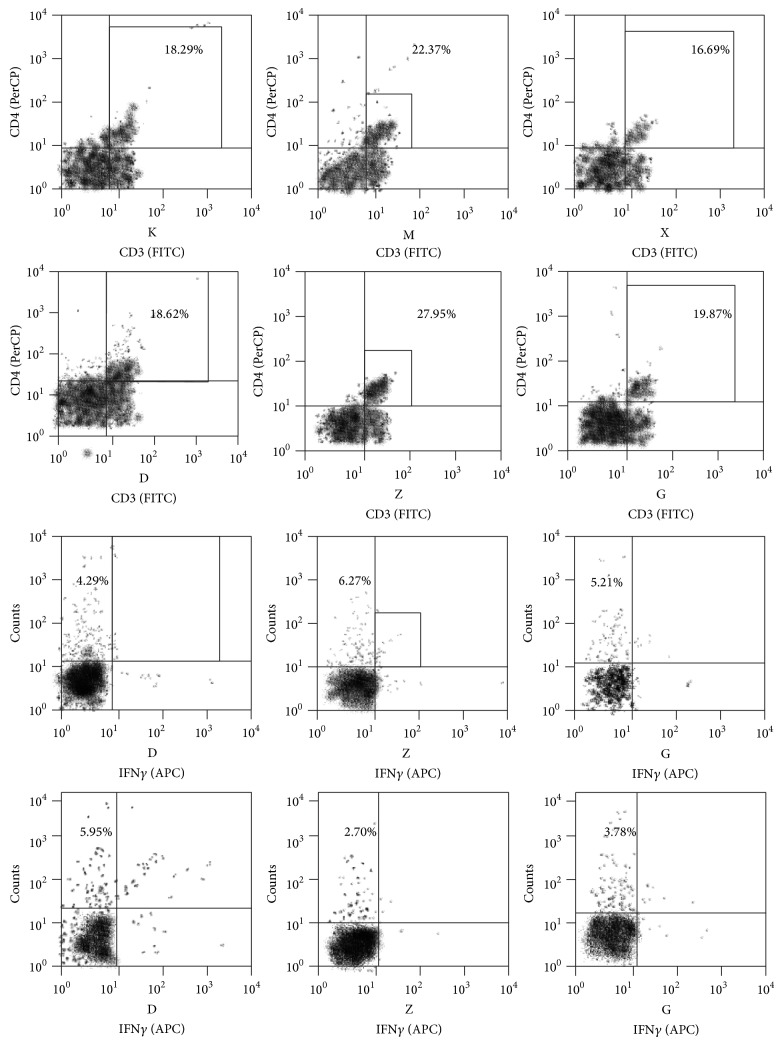
Th17 (CD3+CD4+IL-17+IFN-*γ*−) FACS hepatic tissue for each group.

**Figure 5:**
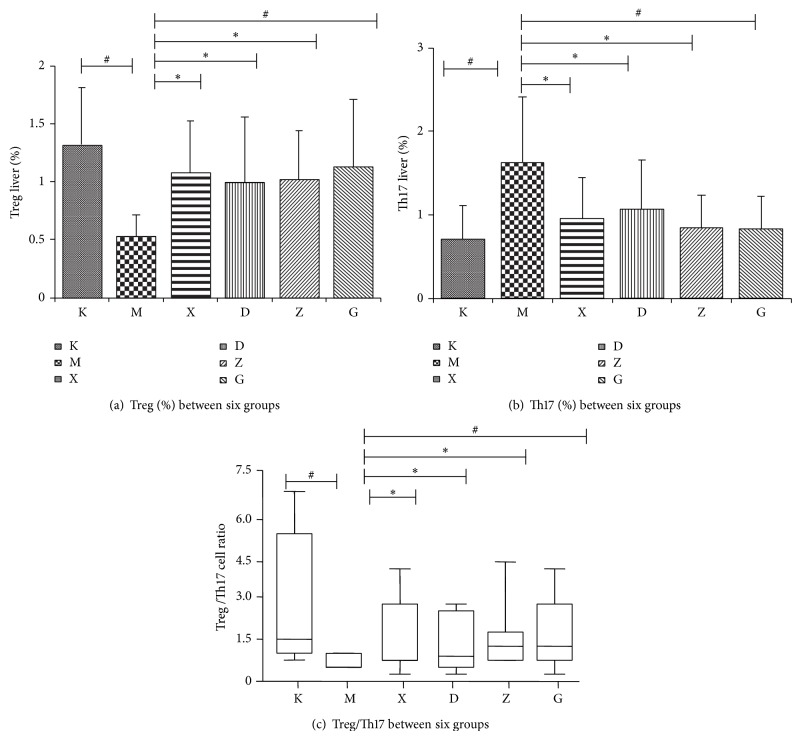
The percentage of Th17 cells and Treg cells and the ratio of the two types of cells.

Analysis results of FACS showed that the Treg cells levels in the hepatic lymph tissue of the model group were lower than in the control group. When each treatment group was compared to the model group, the levels of Treg cells were considerably increased and the difference was statistically significant (*P* < 0.05). Comparison of the control group and the Chinese medicine high dose group to the model group showed a statistically significant difference (*P* < 0.01). The levels of Th17 cells in the model group were higher than in the blank control group and in the treatment groups and the difference was found to be statistically significant (*P* < 0.05). The Treg/Th17 ratio was decreased in the model group when compared to the blank control group. After the use of therapeutic drugs, the ratio of Treg/Th17 was found to be increased. When compared to the model group, the steroids group, Chinese medicine moderate dose group, and Chinese medicine high dose group all had showed increases that were statistically significant (*P* < 0.05). However, there was no statistical difference between the Chinese and steroids groups or between the different Chinese medicine groups. Furthermore, the Chinese medicine treatment group had a dose-dependent trend. These results are shown in Figures 3, 4, and 5 and Tables 2, 3, and 4.

### 3.4. Analysis of Results for Hepatic Tissue Cytokines for Each Group

**Figure 6:**
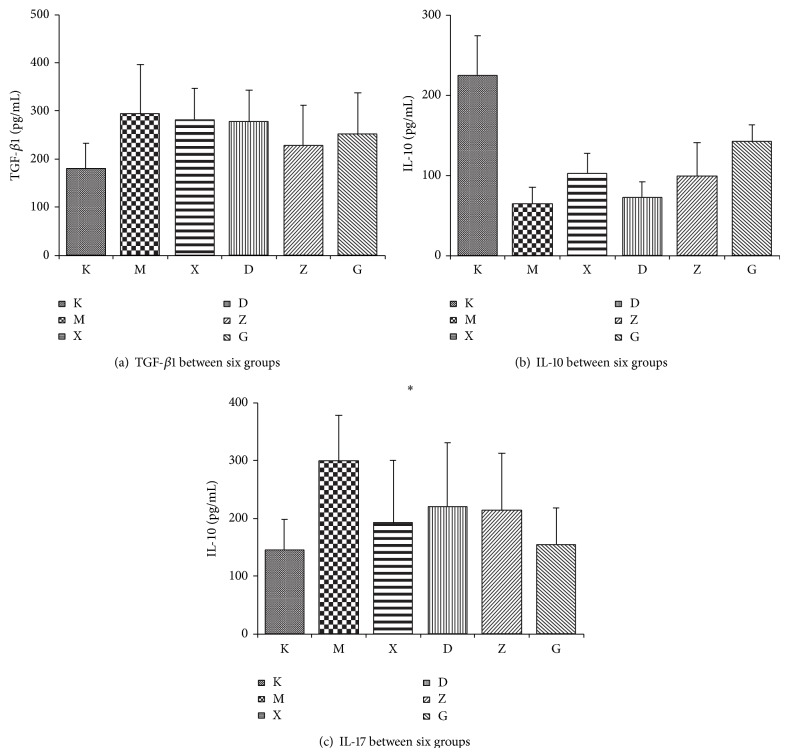
Analysis of hepatic tissue cytokines.

Analysis of TGF-*β*1 in hepatic tissues showed that, in the model group, TGF-*β*1 was increased when compared to the control group but there was no statistical difference between each treatment group and the model group (*P* > 0.05). Analysis of IL-10 showed that model group IL-10 levels were lower when compared to blank control group and when compared to the model group; IL-10 for each treatment group was increased with the difference being statistically significant (*P* < 0.05). Analysis results of IL-17 showed that IL-17 of the model group was considerably higher than that of the blank control group and of each treatment group. The difference was statistically significant (*P* < 0.05). Furthermore, there was no statistical difference between the Chinese medicine and steroids groups or between each of the Chinese medicine groups (*P* > 0.05). Chinese medicine treatment groups showed a dose-dependent trend. Results are shown in Table 4 and Figure 6.

### 3.5. Analysis Results for Liver Specific Transcription Factors Foxp3 and ROR*γ*t

**Figure 7:**
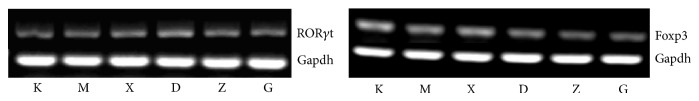
Imaging of Foxp3, ROR*γ*t mRNA gel electrophoresis.

**Figure 8:**
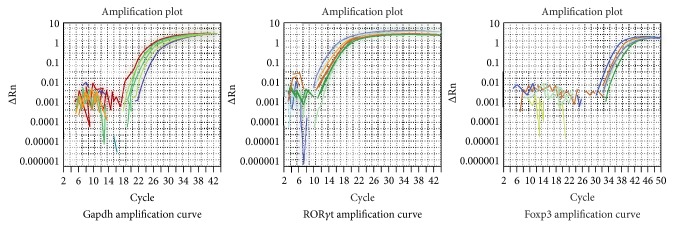
Foxp3, ROR*γ*t mRNA real-time PCR images for each group.

Real-time RT-PCR analysis showed that the difference between each group hepatic tissue transcription factor Foxp3 mRNA was not statistically significant (*P* < 0.05). The model group ROR*γ*t mRNA was higher than that of the control group and treatment groups; the difference was statistically significance (*P* < 0.05, *P* < 0.01). The results are shown in Figures 7 and 8 and Tables 5 and 6.

### 3.6. Results for the Analysis of Hepatic Foxp3 and ROR*γ*t Proteins

**Figure 9:**
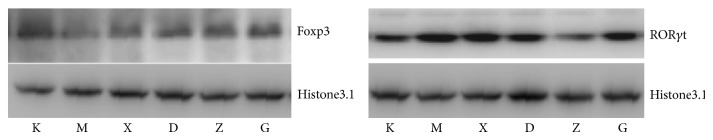
The protein expression of Foxp3 and ROR*γ*t for each hepatic tissue group.

**Figure 10:**
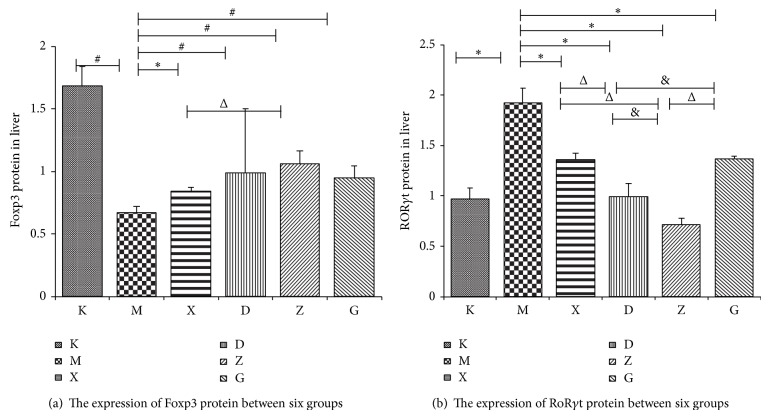
Results for the analysis of hepatic Foxp3 and ROR*γ*t proteins.

For each group, western blot testing showed that Foxp3 protein expression in the model group was much lower than in the control group. In all treatment groups, the expression of the Foxp3 protein was increased when compared to the model group, with the moderate dose Chinese medicine group showing a much higher increase when compared to the steroids group; the difference was statistically significant (*P* < 0.05). The expression of ROR*γ*t protein in the model group was much higher than in the control group. For each treatment group, the ROR*γ*t protein expression was considerably decreased when compared to the model group and the difference was statistically significant (*P* < 0.05). These results are shown in Figures 9 and 10 and Table 7.

## 4. Discussion

Autoimmune hepatitis is a type of autoimmune liver disease which has an undefined etiology and its pathological characteristic is mainly interface hepatitis with infiltration of plasma cells in the portal vein which is accompanied by serum hypergammaglobulinemia and positive serum antibodies [5, 6]. The pathophysiology of AIH is relatively complex with factors such as genetics, autoantigens, environmental factors, and immune dysfunction commonly mentioned as being responsible [7]. Previous studies on the subject mainly focused on liver cell apoptosis, expression of inflammatory mediators, gene polymorphism, and immune system regulation [8–14]. Autoimmune T cells and B cells activation are important steps in the pathophysiology of AIH while the dysfunction of the immunological regulation of the liver has also been shown to be a very important step in the mechanism of the disease [15]. Increasingly, studies are centering their interest on the Treg cell and the Th17 cell and the relationship which exists between these two cells. It is currently thought that loss of balance between these two cells can lead to the induction of the mechanism of abnormal immunity seen in AIH.

*α*-Galcer is able to specifically activate NKT cells, producing several different types of inflammatory cells, activate CD4+ cells, and induce autoimmune hepatitis. After the animal model of the disease was successfully created, pathological analysis showed that the AIH mouse model presented with damaged hepatic cells combined with infiltrated inflammatory cells and regional hepatic cell necrosis. The liver function test indexes, ALT, AST, and AKP, for each group were increased by different degrees and when comparing the control group to the steroids group the difference was statistically significant (*P* < 0.05). Pathological and biochemical indexes showed that our model was able to replicate AIH. After intervention with steroids or Bu Xu Hua Yu treatment, each drug intervention group witnessed a significant decrease in the levels of ALT, AST, and AKP when compared to the AIH model group (*P* < 0.05). Histological studies of hepatic tissues showed different degrees of amelioration which goes to show that Bu Xu Hua Yu Chinese medicine method is able to provide good protection to the liver.

*Treg Cell (Regulatory T Cell) and AIH*. Studies have shown that Treg cells are influenced by IL-10 and TGF-*β*1 during their differentiation, of which the forkhead/winged helix transcription factor, Foxp3, is the specific transcription factor of the Treg cell and, being the main factor of development and cell function effect, it is specifically presented on the Treg cell [16]. Treg cells by way of direct contact inhibit CD8+ T lymph cells proliferation. Their abnormal presentation is a common feature of several autoimmune diseases, for example, systemic lupus erythematosus, rheumatoid arthritis, or multiple sclerosis [17–19]. Furthermore these cells also play a role in the pathophysiology of hepatitis B, hepatitis C, and primary hepatic cancer [20–22]. The newest developments on the study of adult type I AIH have shown that CD4+CD25+Treg cells and NKT cells are considerably decreased when compared to healthy subjects and their products, such as IFN-*γ* and IL-4, are also considerably reduced. This phenomenon has also been observed in infants [23]. In this study, the Treg cell count, IL-10 cytokine levels, and expression of specific transcription factor Foxp3 protein in the model group were decreased when compared to other groups. The expression of TGF-*β*1 was elevated in the model group when compared to other groups; however, the difference was not statistically significant. This could mean that Bu Xu Hua Yu method is able to positively regulate the secretion of IL-10 and Foxp3 and therefore promote the proliferation of Treg cells.

*Th17 Cell and AIH*. Th17 cells following the synergistic action of TGF-*β*1, IL-6, or IL-21 are differentiated from the natural precursor T cells [24], are regulated by specific transcription factor ROR*γ*t [25], and produce the characteristic cytokine interleukin IL-17. These cytokines are involved in host defense, inflammatory diseases, cancer, and transplant rejection reactions. Several studies have reported on the role of Th17 cells and IL-17 in the mechanism of other autoimmune diseases. Th17 cells have been shown to be increased in patients of chronic hepatitis B when compared to healthy people which goes to show that after infection with HBV Th17 has a prominent role after immunological activation [26]. Furthermore, in the mouse model for viral hepatitis, high levels of Th17 cells have also been shown to be present [27]. Collagen-induced arthritis, experimental autoimmune encephalomyelitis, rheumatoid arthritis, inflammatory bowel disease, and systemic lupus erythematosus also show high levels of IL-17 [28–30]. Some researches have discovered that, in Con A-induced AIH animal models, the expression of IL-17a is considerably increased [8] and is mainly concentrated in lymphatic organs or in liver with lymphocytes infiltration. In our study, the Th17 cell count, IL-17 cytokine levels, expression of specific transcription factor ROR*γ*t gene, and protein were all increased in the model group when compared to the control group (*P* < 0.05) and decreased after the use of medication.

The balance between Treg cells and Th17 cells is now known as being the paradigm of the autoimmune field [31]. Even though the exact relation between the loss of balance of the Treg/Th17 ratio in the mechanism of AIH has, as of now, not been clearly determined, however, this phenomenon has also been shown to occur in other diseases such as acute coronary syndrome, myasthenia gravis, non-Hodgkin's lymphoma, or arthritis [32–35]. On the other hand, Longhi et al. have already showed that the inhibition of IL-17 can increase the immunosuppressive activity of Treg cells and thus the promotion of CD25-cells (ngTreg cells) differentiation, while Zhao et al. have shown that IL-17 acts on the expression of hepatic IL-6 which has a direct role in the cause of AIH and according to the same study there exists an opposite relationship between growth, differentiation, and function of Th17 cells and Treg cells [36]. According to Longhi et al. [37], transforming growth factor-*β* is an essential factor in the activation of the differentiation of Th17 cells. However, Fantini et al. have underlined that TGF-*β* and IL-6 are the essential factors for the activation of the differentiation of Th17 cells [38].

This study mainly focuses on the role of the levels of Treg cell and Th17 cell and their function in the pathogenesis of AIH and the loss of balance in the ratio of these two cells in the pathophysiology of AIH. The ratio of Treg cell to Th17 cell was studied and we found that in the control group the ratio was 2.77 and in the model group it was 0.40. After use of medication, the ratio increased. When the model group was compared separately to the control group and to the steroids group, the difference was statistically significant (*P* < 0.05). Therefore, it can be assumed from this study that a loss of balance of the Treg/Th17 cell ratio occurs in AIH and that the level of these two cells and the change in their function has a role to play in the mechanism of AIH. The Bu Xu Hua Yu method can be said to have the same immune suppression role as prednisone.

*Bu Xu Hua Yu Method and AIH*. As of now, the main treatment for AIH is the use of immunosuppressive medications, which is mainly prednisone or low dose of prednisone combined with azathioprine. In addition to this, other immunosuppressants that can be used include cyclosporine A, azathioprine, and KF506; however, these treatment methods do not usually provide the best results because of recurrence, side effects, or incomplete response. Use of western medicine in the treatment of AIH can rarely attain the desired outcome [2].

In our study, the Chinese medicine group was divided into three groups which were low dose, moderate dose, and high dose and we mainly observed the effect of the use of medication on the histology, liver function test, including Treg cell and Th17 cell count and function. The results of this study have shown that the Bu Xu Hua Yu treatment can positively ameliorate AIH in animal models with decrease in the levels of serum ALT, AST, and AKP, causing an alleviation of hepatic inflammation. The results were on par with those obtained with steroids. Different doses of this treatment can increase the expression of IL-10, decrease the expression of IL-17, and regulate the expression of TGF-*β*, which causes the levels of Treg cells to increase and Th17 cell levels to decrease, thus normalizing the Treg to Th17 ratio. In addition to this, the Chinese medicine treatment was also shown to be able to significantly decrease the amount of ROR*γ*t mRNA in hepatic tissues of AIH mice in all groups. This, therefore, negatively influences the role of ROR*γ*t protein on the inhibition of the development and multiplication of Th17 cells, with increase in the expression of Foxp3 and decrease in the expression of ROR*γ*t allowing for the regulation of the development and multiplication of Treg cells and Th17 cells. This process leads to the regulation of the Treg/Th17 cell ratio, which helps in reaching proper functions of immune regulation and immunological protection.

Our study has shown that Chinese traditional medicine components such as Bu Xu Hua Yu method can be used successfully in the preventive treatment of autoimmune hepatitis and can potentially be better tolerated by patients due to Chinese medicine drugs having less side effects. However, additional studies and clinical trials are required to determine the long term effects of using Chinese medicine in the treatment of AIH.

## Figures and Tables

**Table 1:** Liver function indexes for each group.

Group (*n* = 10)	ALT (U/L)	AST (U/L)	AKP (U/L)
K	30.32 ± 9.54^*^	30.9 ± 8.07^*^	35.46 ± 12.16^*^
M	286.16 ± 52.88	253.57 ± 31.46	290.02 ± 59.23
X	186.87 ± 42.65^*^	166.19 ± 33.88^*^	147.3 ± 38.25^*^
D	192.22 ± 44.32^*^	170.63 ± 26^*^	179.86 ± 37.85^*^
Z	186.05 ± 40.79^*^	165.60 ± 22.41^*^	168.89 ± 20.25^*^
G	156.99 ± 34.59^*^	147.64 ± 25.83^*^	137.86 ± 45.92^*^

When compared to the model group, ^*^ *P* < 0.05.

**Table 2:** Each group hepatic tissues Treg, Th17 cell levels (%).

Group (*n* = 8)	Foxp3(+) Treg	TH17 (IL17+IFN*γ*−)
K	1.32 ± 0.49^#^	0.71 ± 0.40^#^
M	0.53 ± 0.18	1.63 ± 0.78
X	1.08 ± 0.44^*^	0.95 ± 0.49^*^
D	1.00 ± 0.56^*^	1.07 ± 0.59^*^
Z	1.02 ± 0.42^*^	0.84 ± 0.39^*^
G	1.13 ± 0.58^#^	0.83 ± 0.39^#^

^*^When compared to M, *P* < 0.05;  ^#^when compared to M, *P* < 0.01.

**Table 3:** Treg/Th17 cell ratio for each group.

Group (*n* = 8)	Treg/TH17	Mean rank	When compared to M group *Z* ratio
K	2.77 ± 2.57^*^	34.38	3.37
M	0.40 ± 0.24	8.38	—
X	1.64 ± 1.35^*^	26.5	2.95
D	1.41 ± 1.26	23.63	1.90
Z	1.58 ± 1.35^*^	26.38	2.74
G	1.72 ± 1.37^*^	27.75	2.63

^*^When compared to M, *P* < 0.0083.

**Table 4:** Analysis results for cytokines in hepatic tissues for each group.

Group (*n* = 10)	TGF-*β*1 (pg/mL)	IL-10 (pg/mL)	IL-17 (pg/mL)
K	179.36 ± 53.57^*^	224.47 ± 49.59^*^	145.23 ± 52.49^*^
M	293.82 ± 102.18	64.5 ± 20.25	298.71 ± 79.50
X	281.79 ± 64.70	102.45 ± 24.80^*^	192.29 ± 107.56^*^
D	277.14 ± 64.86	72.55 ± 18.73^*^	219.95 ± 110.76^*^
Z	228.71 ± 83.21	99.37 ± 41.63^*^	213.42 ± 98.50^*^
G	251.36 ± 85.11	141.68 ± 21.60^*^	154.74 ± 62.96^*^

When compared with group M, ^*^ *P* < 0.05.

**Table 5:** Results for the analysis Foxp3 mRNA for each group (2-ΔΔCT).

Group (*n* = 8)	RQ (2-ΔΔCT) median	RQ (2-ΔΔCT) Q25	RQ (2-ΔΔCT) Q75	*Z* ratio	*P* ratio
K	0.1887^#^	0.1167	1.9460	2.731	0.006
M	0.0529	0.0121	0.1038	—	—
X	0.0729	0.0332	0.1886	1.470	0.141
D	0.0842	0.0549	0.1391	1.470	0.141
Z	0.1152	0.0432	0.4352	1.575	0.115
G	0.0642	0.0181	0.2824	0.840	0.401

When compared to group M, ^#^ *P* < 0.05.

**Table 6:** Results for the analysis of ROR*γ*t mRNA for each group (2-ΔΔCT).

Group (*n* = 8)	RQ (2-ΔΔCT)	*P* ratio
K	0.110 ± 0.062	0.01^*^
M	0.393 ± 0.282	—
X	0.201 ± 0.146	0.020^*^
D	0.134 ± 0.075	0.002^*^
Z	0.161 ± 0.122	0.005^#^
G	0.165 ± 0.085	0.006^#^

When compared to group M, ^*^ *P* < 0.05 and ^#^ *P* < 0.01.

**Table 7:** Foxp3, ROR*γ*t proteins for each liver group.

Group (*n* = 8)	ROR*γ*t protein (IOD ratio)	Foxp3 protein (IOD ratio)
K	0.97 ± 0.11^#^	1.68 ± 0.16^#^
M	1.92 ± 0.15	0.67 ± 0.05
X	1.36 ± 0.06^#^	0.84 ± 0.03^*^
D	0.99 ± 0.13^#Δ^	0.99 ± 0.51^#^
Z	0.71 ± 0.07^#Δ&^	1.06 ± 0.10^#Δ^
G	1.37 ± 0.02^#&^	0.95 ± 0.09^#^

^*^When compared with group M, *P* < 0.05 and ^#^ *P* < 0.01;  ^Δ^when compared with group X, *P* < 0.05;  ^&^when compared with group D, *P* < 0.01.
